# Perineural Invasion in Cervical Cancer: A Hidden Trail for Metastasis

**DOI:** 10.3390/diagnostics14141517

**Published:** 2024-07-14

**Authors:** Guoqiang Chen, Hao Sun, Yunxia Chen, Li Wang, Ouyi Song, Jili Zhang, Dazhi Li, Xiaojun Liu, Lixia Feng

**Affiliations:** 1 Department of Obstetrics and Gynecology, Second Affiliated Hospital of Naval Medical University, Shanghai 200003, China; 2 Department of Gynecology, The People’s Hospital of Baoan Shenzhen, The Second Affiliated Hospital of Shenzhen University, Shenzhen 518101, China

**Keywords:** cervical cancer, perineural invasion, Type C1 surgery, Schwann cells, neurotrophic factors

## Abstract

Perineural invasion (PNI), the neoplastic invasion of nerves, is an often overlooked pathological phenomenon in cervical cancer that is associated with poor clinical outcomes. The occurrence of PNI in cervical cancer patients has limited the promotion of Type C1 surgery. Preoperative prediction of the PNI can help identify suitable patients for Type C1 surgery. However, there is a lack of appropriate preoperative diagnostic methods for PNI, and its pathogenesis remains largely unknown. Here, we dissect the neural innervation of the cervix, analyze the molecular mechanisms underlying the occurrence of PNI, and explore suitable preoperative diagnostic methods for PNI to advance the identification and treatment of this ominous cancer phenotype.

## 1. Introduction

**Figure 1:**
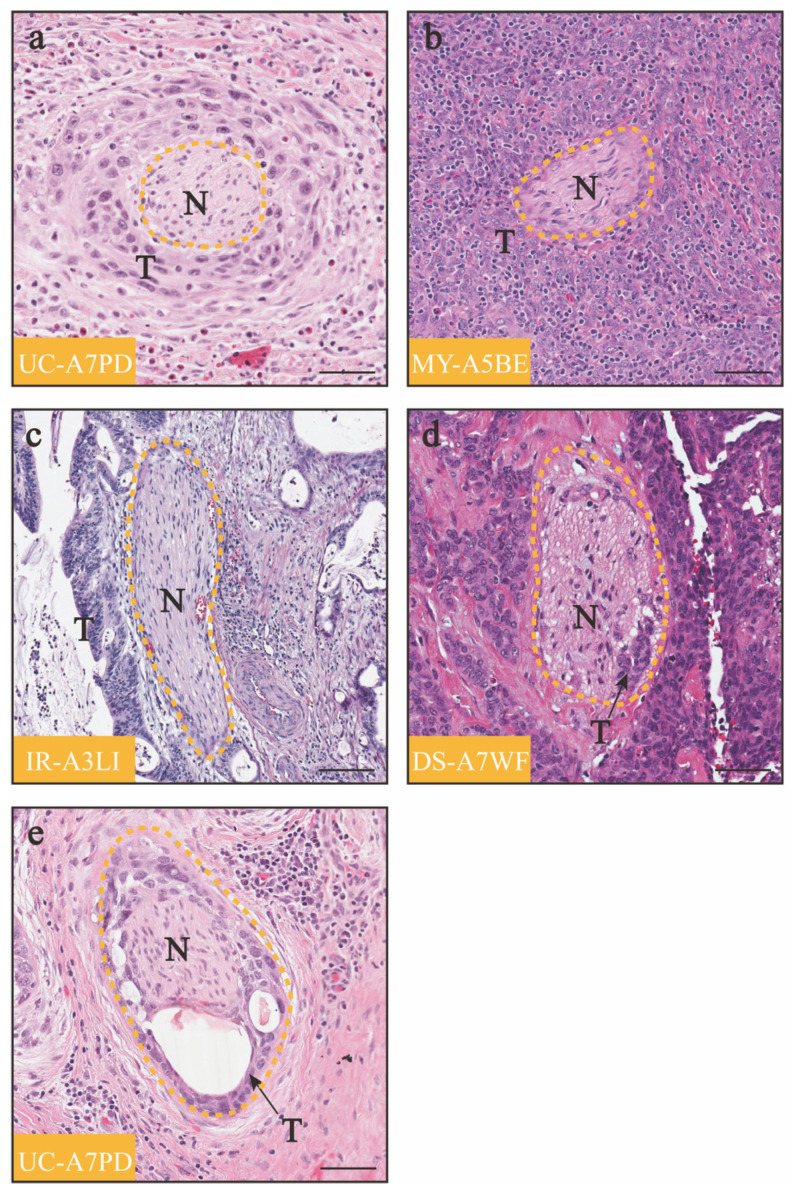
Variety of histologic appearance patterns of PNI in cervical cancer patients from the TCGA cohort. (**a**) Tumor cells encircle the nerve outside the main tumor bulk. (**b**) Nerve is surrounded by tumor cells inside the main tumor bulk. (**c**) Tumor cells surround at least 33% of the nerve circumference. (**d**) Tumor cells invade the nerve. (**e**) Tumor cells invade the nerve and disrupt the integrity of the nerve. “N” represents nerves, “T” represents tumor cells, and the yellow dotted lines show the structure of the nerve; scale bar: 200 μm.

Nerves are emerging participants in tumor progression. Cancer cells induce the outgrowth of nerves in the tumor microenvironment (TME) through the release of neurotrophic factors. In turn, nerves release neurotransmitters that activate cancer growth and dissemination while also influencing the local environment by regulating angiogenesis and the immune system [1,2]. Research across various cancer types indicates a close association between higher intratumoral neural density and an unfavorable cancer prognosis [3,4]. For example, the presence of innervation in the TME has been found to be associated with tumor aggressiveness in conditions such as salivary adenoid cystic carcinoma [5], breast cancer [6], pancreatic cancer [4], gastric cancer [7], prostate cancer [8], and ovarian cancer [9]. Another mechanism through which the nervous system supports the migration of tumor cells is through perineural invasion (PNI), a process involving the invasion and migration of cancer cells along nerve sheaths. PNI refers to the involvement of tumor cells in more than one-third of the circumference of a nerve or the infiltration of any layer of the three-layer nerve sheath [10] (Figure 1). PNI manifests as a bidirectional tropism between nerves and cancer cells, orchestrated by the expression of specific molecules on both the tumor cells and surrounding peripheral nerves. Once PNI occurs, the affected nerves become sanctuaries for tumor cells. These tumor cells utilize this neural structure as a pathway for metastasis, avoiding clearance by the body and resistance to chemotherapy drugs [11,12]. Research has shown that the incidence of PNI in pancreatic ductal adenocarcinoma can reach 100% when enough pathological tissue sections are carefully analyzed [13,14]. In contrast, the incidence of PNI in cervical cancer patients ranges from only 7.0% to 35.1% [15]. However, cervical cancer patients with PNI also exhibit decreased survival [16]. Due to the propensity for PNI in cervical cancer, there is a consensus among experts that when a cervical biopsy or cervical conization indicates a relevant PNI, it should be considered a contraindication for Type C1 surgery [17]. In this review, our aim is to summarize the current literature on innervation and the PNI in cervical cancer. We focused on the clinical significance of PNI in cervical cancer patients and reviewed the molecular mechanisms underlying its occurrence.

## 2. Evolution of Surgical Classifications for Cervical Cancer

In 1898, the Austrian surgeon Wertheim performed the world’s first abdominal radical hysterectomy for cervical cancer [18]. In 1974, Professor Piver proposed a five-type classification system for cervical cancer surgery [19]. Subsequently, in 2008, the Querleu–Morrow classification (Q–M classification) for radical hysterectomy was introduced [20]. In 2015, the National Comprehensive Cancer Network (NCCN) guidelines recommended shifting from the Piver classification to favor the Q–M classification [21]. In 2017, the Q–M classification underwent a revision [22] by incorporating the Cibula 3-D concept [23], reflecting the latest anatomical concepts and encompassing new approaches to almost all cervical cancer surgeries. The Q–M classification divides the scope of uterine resection into A, B, C, and D types, with subtypes B1, B2, C1, C2, D1, and D2. The C type, which involves extensive uterine resection, serves as the fundamental procedure for cervical cancer surgery. Based on whether the pelvic autonomous nerves are preserved, the C type is further classified into Type C1 (nerve-sparing radical hysterectomy, NSRH) and Type C2 (radical hysterectomy without preserving autonomic nerves, RH). Conventional extensive uterine resection can cause varying degrees of damage to pelvic autonomous nerve structures, leading to postoperative complications such as bladder dysfunction, rectal dysfunction, and sexual dysfunction. Clinical studies have confirmed that Type C1 surgery and Type C2 surgery are equally effective in treating cervical cancer [24,25], and there is no significant difference in patient prognosis [24,26,27]. To improve the postoperative quality of life for patients, the 2017 Q–M classification recommends promoting Type C1 surgery as the primary procedure for radical treatment of cervical cancer worldwide [22]. It is worth noting that cervical cancer exhibits a trend toward PNI. Therefore, PNI is a contraindication for Type C1 surgery. Chinese experts also recommend implementing Type C1 surgery for stage IB tumors with a diameter ≤4 cm, without deep stromal invasion of the cervix, without lymphovascular space invasion, without vaginal involvement, and without risk factors for lymph node metastasis [17].

## 3. Anatomic Mapping of Uterine Innervation

**Figure 2:**
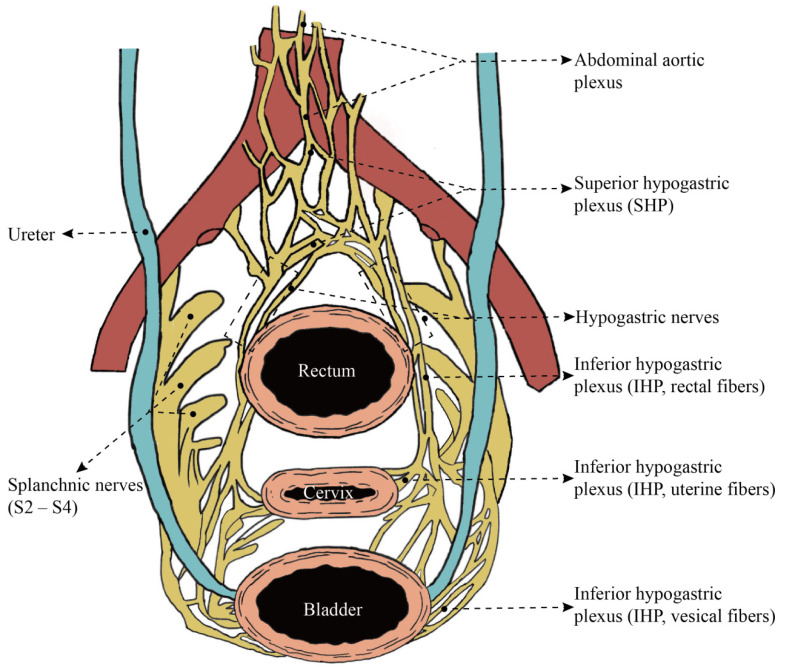
Pelvic splanchnic nerves. Scheme of female pelvic autonomic nerves, overhead projection.

The superior hypogastric plexus (SHP) and hypogastric nerves are the main sources of sympathetic innervation to the pelvic organs. The SHP is formed by the continuation of the abdominal aortic plexus, located below the abdominal aortic bifurcation, in the region between the two common iliac arteries [28]. Its lower end extends approximately to the level of the sacral promontory, where it bifurcates into paired hypogastric nerves [28,29]. The left and right hypogastric nerves extend downward to the pelvic floor, on the medial side of the ureter and the lateral side of the uterosacral ligament in Okabayashi’s space [28,30], and combine with splanchnic nerves from S_2_ to S_4_ to form the inferior hypogastric plexus (IHP) [28,29]. The IHP sends out branches to supply the physiological functions of the rectum, uterus, vagina, and bladder, including the rectal fibers, uterine fibers, vaginal fibers, and vesical fibers (Figure 2). Among the areas of the human uterus, the nerve fibers of the cervix are the most densely distributed [31,32,33]. Specifically, the cervix exhibits a preference for certain types of nerves, with 79% TH+ autonomic sympathetic fibers, 73% NPY+ sensitive fibers, and 13% VIP+ parasympathetic fibers [32]. The abundant neural innervation results in the highest concentration of almost all neurotransmitters in the uterine cervix compared to the fundus and uterine body [31,33]. These neurotransmitters are involved in pain, vascular formation, and the constriction of pregnancy and childbirth [33]. For cervical cancer patients eligible for Type C1 surgery, excision should be carried out close to the organ to avoid damaging the IHP located on both sides of the organ, thus minimizing the risk of postoperative organ dysfunction.

## 4. Clinical Studies of Cervical Cancer PNI

Several studies have consistently shown that cervical cancer patients with PNI have a lower overall survival (OS) rate [16,34,35,36,37,38,39], along with a decrease in disease-free survival (DFS) [35,37,38,39] or progression-free survival (PFS) [36] (Table 1). Compared to patients in the matched group, PNI (+) patients had advanced clinical stage [40,41,42,43,44], advanced tumor grade [40], increased tumor size [37,40,41,43], deep cervical stromal invasion [35,36,37,39,40,41,42,44], parametrial invasion [36,37,39,43,44], lymphovascular space invasion [35,37,39,40,43,44], positive lymph nodes [35,36,37,39,40,44], and positive margins [35,36,44]. Moreover, PNI (+) cervical cancer patients were more likely to receive adjuvant therapy after surgery [34,36,37,39,40,43,44] (Table 1). Therefore, the PNI can be regarded as a poor prognostic factor for patients with cervical cancer. However, in the studies reported by Karim S. Elsahwi (PNI was 12.5%, 24 out of 192 patients) [43] and Hyun Chul Cho (PNI was 7%, 13 out of 185 patients) [44], no differences in DFS or OS were observed based on the presence of PNI. Compared to the study by Wan Ting (PNI was 8.8%, 162 out of 1836 patients; possibly the largest study of cervical cancer patients with PNI among published data) [36], the sample sizes of Karim S. Elsahwi and Hyun Chul Cho appear to be relatively small and may leave out a small number of patients with an insignificant PNI. In detail, the study by Wan Ting shows the 3-year PFS of PNI (+) cervical cancer patients and the matched group were 76.4% and 87.8%, respectively, whereas the 3-year OS was 78.6% and 93.9% [36].

## 5. Preoperative Assessment Methods

### 5.1. Biopsy

Preoperative PNI prediction might help identify populations that could obtain maximum benefits from Type C1 surgery without compromising oncologic safety. Traditionally, the identification of PNI primarily relies on postoperative pathological hematoxylin and eosin (H&E) staining and immunohistochemistry (IHC). However, the inconspicuous PNI status can be easily obscured under HE staining, and nerves severely infiltrated by tumor cells are also challenging to distinguish under HE staining. This often leads to underdiagnosis in PNI detection. Therefore, enhancing the visualization of the PNI becomes clinically significant. IHC examination of S100 neurospecific proteins can assist in diagnosing PNI. S100 is primarily expressed in Schwann cells (SCs) of nerve bundles, and immunohistochemical staining distinctly highlights nerve bundles, aiding in the identification of their location, quantity, and structural relationship with tumor cells [45,46,47,48,49]. In addition, when cancer cell clusters spread around or infiltrate nerves, distinguishing them from inflammatory cells also becomes a challenging task. In doubtful cases, double immunohistochemical staining of S100 and AE1/AE3 can improve the detection of small nerves invaded by diffuse gastric cancer [50], skin cancer [51], and vulvar cancer [52,53]. Double immunohistochemical staining has more advantages than H&E staining in the definitive diagnosis of PNI. However, performing rapid frozen section examination and immunohistochemistry intraoperatively is impractical. How can such a large piece of tumor tissue be rapidly located to identify affected nerve fibers? Furthermore, completing IHC staining within one hour is even more unlikely. Cervical biopsy and cervical conization pathologic results are also limited in guiding surgery, as they generally represent only a small part of the cervical tissue and may not include nerves. Therefore, a “negative” result may not rule out the possibility of pelvic nerve involvement. Some scholars have proposed performing a laparoscopic biopsy of uterine nerve branches a few days before surgery to develop surgical plans [54]. It is also impractical, as no patient would be willing to undergo additional surgery in terms of both physical and financial costs. 

### 5.2. Imaging Methods

The 2018 FIGO staging for cervical cancer has moved beyond sole reliance on clinical staging, introducing a comprehensive evaluation approach that combines clinical examinations, imaging studies, and pathological assessments. Modern techniques featuring high-resolution capabilities are being explored as potential components of noninvasive PNI diagnostics [55]. Capek and colleagues reported instances of visualizing the PNI using MRI, FDG/PET CT, and choline PET/CT [56]. Their findings suggested that affected nerves commonly display enlargement with irregular and nodular contours on T1-weighted sequences, while T2-weighted images show hyperintensity [56]. These developments indicate a promising direction for the use of advanced imaging techniques for PNI assessment in cervical cancer and other pelvic malignancies. Ziv Gil and colleagues demonstrated in an animal model that intraoperative fluorescent stereoscopic imaging showed a significantly enhanced eGFP signal in a mouse PNI model treated with NV1066, identifying the affected nerves that needed to be excised [57]. However, this method is currently limited to the animal level, and its clinical application still has a long way to go.

### 5.3. Predictive Nomogram

Recently, some studies have focused on nomograms to establish risk assessment models for predicting the tumor PNI based on preoperative indicators, aiding in the formulation of more rational and accurate preoperative surgical plans [58,59,60]. Wan Ting et al. constructed a nomogram including age, adenocarcinoma, tumor size, neoadjuvant chemotherapy, lymph node enlargement, deep stromal invasion, and full-layer invasion to predict the PNI status in cervical cancer patients (AUC of 0.763 for the training set, AUC of 0.860 for the validation set, and AUC of 0.915 for the revised validation set). AUC is the area under the receiver operating characteristic curve [61]. It has satisfactory predictive performance and can help identify cervical cancer patients with false-negative PNI results. Although further large-scale, multicenter studies are needed to validate the predictive nomogram before its extensive use, it demonstrates significant practical advantages compared to biopsies. 

## 6. Mechanisms of PNI in Cervical Cancer

### 6.1. “Defects” of the Peripheral Nerve Sheaths

The peripheral nerve sheath is composed of the epineurium, perineurium, and endoneurium. The epineurium is connective tissue that surrounds the surface of the nerve, and its surface is formed by several layers of flat epithelial cells. A nerve contains multiple nerve fiber bundles, and the thin layer of connective tissue on the surface of these fiber bundles is called the endoneurium. The extensive connective tissue between the epineurium and endoneurium forms the perineurium. Some scholars believe that the structure of peripheral nerves is a continuation of the development of spinal nerve structures, with the endoneurium, perineurium, and epineurium corresponding to the pia mater, arachnoid mater, and dura mater, respectively [62]. There are potential gaps between them that facilitate the infiltration and spread of cancer cells, creating so-called “low-resistance channels” [62,63]. Because nerves, blood vessels, and lymphatics often travel together, the blood-nerve barrier confers a privileged status to the endoneurial compartment. When hematogenous spread occurs, infiltration into the endoneurium likely occurs through direct spread from the epineurium [64]. However, lymphatic channels do not penetrate the inner parts of the nerve sheath [10,64], so the occurrence of PNI may be associated with hematogenous spread but not with lymphatic metastasis.

### 6.2. Neurotrophic Factors

Neurotrophic factors secreted by neuronal cells and cancer cells play a crucial role in the field of tumor neuroscience. Neurotrophic factors are divided into three main families: neurotrophins (NTs), ciliary neurotrophic factors (CNTFs), and glial cell-derived neurotrophic factors (GDNFs). The NT family is well characterized and consists of five members: nerve growth factor (NGF), brain-derived neurotrophic factor (BDNF), neurotrophin 3 (NT-3), neurotrophin 4/5 (NT-4/5), and neurotrophin 6 (NT-6). NTs interact with two types of receptors: p75 and Trk receptors. The p75 receptor exhibits a low-affinity binding capacity for all NTs. Conversely, Trk receptors display high-affinity binding specificity for different NTs: NGF binds to TrkA, BDNF and NT4/5 bind to TrkB, and NT-3 binds to TrkC [65]. When nerves are injured, SCs can promote the survival and repair of damaged neurons and axons by secreting neurotrophic factors such as NGF, BDNF, NT-3, and GDNF [66,67]. Similarly, within the tumor microenvironment, there is a process of neural repair similar to that observed during nerve damage [68]. This concept evokes the analogy of comparing a tumor to a wound that never heals [69].

**Figure 3:**
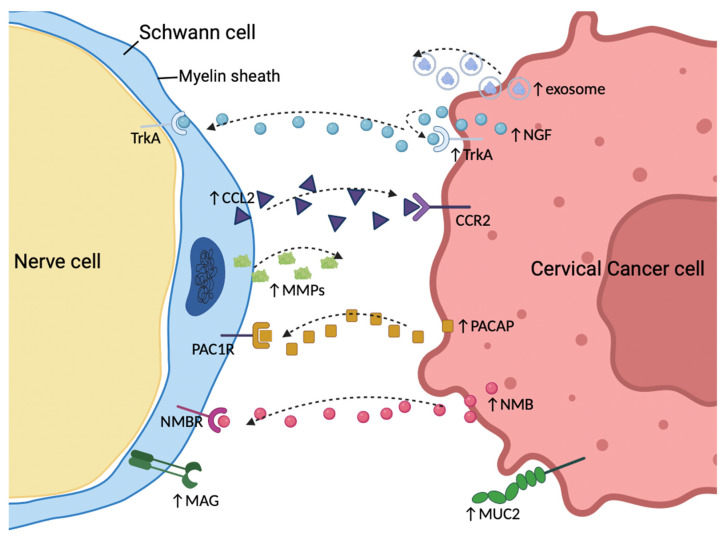
Signaling molecules are involved in the process of perineural invasion in cervical cancer. Interactions between molecules expressed on cancer cells and peripheral nerves play an important role in perineural invasion (PNI). Cancer-derived exosomes and nerve growth factor (NGF) have been shown to be relevant for tumor innervation and PNI. Schwann cells can secrete CCL2, which acts on its corresponding receptor, CCR2, thus promoting tumor migration and invasion. In turn, cervical cancer cells can reverse the phenotype of Schwann cells to trigger PNI by secreting PACAP and NMB. Transmembrane proteins, such as MUC2 and MAG, may also facilitate tumor-nerve adhesion, providing a potential route for PNI. Additionally, matrix metalloproteinases (MMPs) secreted by Schwann cells could pave the way for tumor cell metastasis. PNI, perineural invasion; NGF, nerve growth factor; PACAP, pituitary adenylate cyclase-activating polypeptide; NMB, neuromedin B; MUC2, mucin 2; MAG, myelin-associated glycoprotein; MMPs, matrix metalloproteinases.

Ying Long et al. found that high levels of NGF and TrkA expression correlate with the PNI in early-stage cervical cancer [70]. NGF can induce the proliferation and migration of cervical cancer cells [71]. Moreover, cervical cancer cell proliferation has been identified as a common phenomenon during PNI [72], establishing a basis for cancer cell colonization and metastasis along nerves. NGF secreted by cancer cells can additionally promote axon guidance and recruit nerves [73,74], thereby facilitating PNI in pancreatic cancer. Although studies indicate that exosomes secreted by cervical cancer cells induce their own innervation [75], it remains unclear whether NGF is packaged within exosomes or is required for exosome-mediated neurite outgrowth (Figure 3). Tumor-derived BDNF promotes increased innervation and is associated with poor outcomes in patients with ovarian cancer [9]. Furthermore, SCs facilitate the PNI process in salivary adenoid cystic carcinoma through the BDNF/TrkB axis [76]. Although there is currently no direct evidence of BDNF involvement in the neural innervation or PNI of cervical cancer, experiments have indicated that BDNF can enhance the motility and anoikis resistance of cervical cancer cells [77,78]. High expression of BDNF is also positively correlated with advanced FIGO stage, lymph node metastasis, and a poor prognosis in cervical cancer patients [79,80]. Therefore, BDNF may also play a crucial role in the PNI of cervical cancer patients.

### 6.3. Chemokines

The aberrant release of chemokines is closely associated with PNI in cancer [14,81]. Nerve-released CCL2 supports the migration and PNI of prostate cancer through CCR2-mediated signaling [82]. Further research by Richard L. Bakst et al. revealed that SCs secrete CCL2, which recruits inflammatory monocytes from the circulation via CCR2, leading to the production of cathepsin B in these monocytes and ultimately disrupting the perineurium [83]. As expected, CCL2 is also a significant participant in PNI in cervical cancer. SCs migrate to the vicinity of the tumor and secrete CCL2 under signals derived from cervical cancer cells, which acts as a potent chemoattractant, inducing CCR2+ cervical cancer cells to undergo epithelial–mesenchymal transition (EMT) and move along neurites. In turn, signals from cancer cells trigger the expression of matrix metalloproteinases (MMPs) in SCs, facilitating extracellular matrix dissolution along the axon [84] (Figure 3).

### 6.4. Schwann Cells

Schwann cells (SCs) are glial cells in the peripheral nervous system, and their remarkable plasticity enables regeneration of the peripheral nervous system after injury [67]. In this process, SCs undergo two major changes. One of these effects is the upregulation of various neuropeptides that support the survival and axonal elongation of injured neurons. Among these neuropeptides are galanin, substance P (SP), and calcitonin gene-related peptide (CGRP), among others [85]. Neuropeptides are typically expressed at low levels in peripheral nerves but increase following injury and inflammation. A study revealed that following injury or inflammation, neurons release galanin, which activates head and neck cancer to favor PNI; in turn, the release of galanin from head and neck cancer induces neuritogenesis [86]. Moreover, when nerves are invaded by tumor cells, the nerves produce more galanin, thereby enhancing nerve–tumor interactions [86]. Similarly, neurogenic SP and CGRP can enhance the malignant phenotype of cancer cells, contributing to PNI [87,88,89].

Another component of SCs in the injury response is the reversal of myelin differentiation. The demyelination of SCs results in extensive cellular elongation and branching, generating a distinctive repair cell morphology that is favorable for the formation of regeneration tracks [90]. Molecules that characterize demyelinated SCs, including vimentin, nestin, c-Jun, and glial fibrillary acidic protein (GFAP), are upregulated [67]. Notably, when cervical cancer cells were cocultured with SCs, SCs were initially activated and migrated toward cervical cancer before the onset of cancer invasion [84,91]. At the same time, when cocultured with cervical cancer cells, the SCs exhibited an upregulation of demyelination markers, such as GFAP, vimentin, and nestin [91]. Based on these results, a new doctrine is further reinforced: it may be nerves—and not cancer cells—that first attack each other [92,93,94]. Through immunofluorescence staining, our previous research revealed that SCs migrate into the main bulk of the tumor and integrate into the intercellular spaces among cervical cancer cells [91]. This further indicates that signals from cervical cancer cells trigger Schwann cells, making them the precursor cells for PNI in cervical cancer. This interaction may be mediated by cervical cancer-derived pituitary adenylate cyclase-activating polypeptide (PACAP) [91]. Moreover, research by Ting Huang demonstrated that neuromedin B (NMB), derived from cervical cancer cells, triggers the reprogramming of SCs by binding to its membrane receptor NMBR, which in turn promotes axonal regeneration and induces PNI [95] (Figure 3).

### 6.5. Mucin

During PNI in pancreatic cancer, cancer cells that overexpress membrane-bound mucin 1 (MUC1) can establish strong adhesion with myelin-associated glycoprotein (MAG) on Schwann cells [96]. This selective adhesive advantage facilitates the invasion of cancer cells into the endoneurium of nerves. MUC1 also enhances the invasiveness of pancreatic cancer cells by inducing EMT [97], which may also contribute to the occurrence of PNI. In our previous research [91], we identified elevated expression levels of mucin 2 (MUC2) in PNI (+) cervical cancer patients in the TCGA cohort through the evaluation of perineural invasion and differential expression analysis (log2 fold-change = 3.15, *p* < 0.001; Figure 3; a volcano plot is shown in Supplementary Figure S1). MUC2 is a major mucin primarily secreted by goblet cells that forms a protective barrier on the surface of the intestinal epithelium and plays a crucial role in colonic protection [98]. Notably, both increased and decreased expression of MUC2 are associated with cancer progression [99,100,101,102,103,104]. Whether MUC2 promotes PNI in cervical cancer through selective adhesion and EMT or by generating a mucous barrier to protect cancer cells from recognition by antitumor immune effectors is not yet known. Our research team is actively exploring this issue.

## 7. PNI in Other Gynecological Cancers

Firstly, neural activities play a vital role in ovarian cancer progression, among which axon guidance is particularly significant [105]. Sustained adrenergic signaling has been associated with increased tumoral innervation in ovarian cancer [9], and elevated tumor nerve counts may also foster the occurrence of PNI. Zheng Zhen et al. further confirmed that the PNI is a strong predictor of poor prognosis in patients with ovarian cancer [106]. Nonetheless, the mechanisms that initiate PNI in ovarian cancer need further exploration. Secondly, the incidence of PNI in patients with vulvar cancer is approximately 30% [52,107]. Patients with PNI (+) vulvar cancer exhibit significantly shorter DFS and OS [52,107,108,109], indicating that the PNI should be regarded as an independent adverse prognostic factor for an unfavorable course of vulvar cancer [52,53,108,109,110]. However, the underlying mechanisms of PNI occurrence in vulvar cancer remain to be elucidated. Finally, the release of glutamic acid by neurons could promote PNI in endometrial carcinoma through the activation of GluR2 [111].

## 8. Conclusions

With the standardization and promotion of screening techniques, an increasing number of cervical cancer cases are being detected at an early stage. RH remains the primary treatment option for early-stage cervical cancer, but it has been shown to result in pelvic autonomous nervous system dysfunction, including lower urinary tract dysfunction and sexual dysfunction, significantly reducing patients’ quality of life. Therefore, Type C1 surgery has been used to preserve the pelvic autonomous nerves. However, due to the presence of PNI in cervical cancer patients, the implementation of Type C1 surgery has been limited. Despite the dense innervation of the cervix, the prevalence of PNI in cervical cancer patients remains low, and the reasons for this phenomenon are not yet fully understood. Because of the low incidence of PNI in cervical cancer patients, it has not previously received sufficient attention from surgeons. However, existing clinical studies have consistently shown that PNI (+) cervical cancer patients have a poor prognosis, which compels gynecologic oncologists to no longer ignore this aspect. Hence, studying PNI in cervical cancer is not about negating Type C1 surgery but rather about better clarifying the surgical indications of this procedure and more perfectly demonstrating its superiority for both patient survival and improving quality of life.

PNI is often underreported due to the lack of standardized reporting standards. Specific immunohistochemical staining of SCs using S100, in sharp contrast to counterstaining with HE, allows for a rapid and straightforward improvement in the detection rate of PNI. However, the challenges of specimen collection and the time-consuming staining process mean that its utility in guiding preoperative and intraoperative surgical planning is still limited. Imaging evaluation is also challenging for the timely identification of early PNI. Nomograms, which serve as a clinical tool to predict clinical events and outcomes, show promise for predicting the PNI in cervical cancer patients. We believe that a predictive nomogram can be considered one of the optimal solutions for the preoperative evaluation of PNI in cervical cancer patients.

Regrettably, there is limited research on the mechanism of PNI occurrence in cervical cancer. However, existing clinical studies have highlighted the significant role of SCs in this process. Inflammation accompanies cancer, acting as a catalyst for the progression of cancer development [112,113]. Therefore, neurons and SCs in the inflammatory tumor environment can promote the migration and invasion of tumor cells by secreting chemokines. Notably, the latest viewpoint suggests that tumor cells can actively secrete neuropeptides and other molecules to reverse the phenotype of SCs, thereby hijacking SCs to assist in their own neural metastasis. In conclusion, exploring the potential pathological mechanisms of PNI in cervical cancer through SCs will be significant for the surgical selection and treatment of cervical cancer.

## Figures and Tables

**Table 1:** Association between the PNI and survival and clinicopathological features in cervical cancer patients.

Reference	Number and Incidence	OS	DFS or PFS	Advanced Clinical Stage	Advanced Tumor Grade	Increased Tumor Size	Deep Cervical Stromal Invasion	Parametrial Invasion	Lymphovascular Space Invasion	Positive Lymph Nodes	Positive Margins	More Adjuvant Therapy
Horn, L.C. 2010 [16] Meinel, A. 2011 [42]	35.1% (68/194)	OS ↓	NS	+	NS	/	+	/	/	/	/	/
Elsahwi, K.S. 2011 [43]	12.5% (24/192)	NS	NS	+	NS	+	NS	+	+	NS	NS	+
Cho, H.C. 2013 [44]	7% (13/185)	NS	NS	+	/	NS	+	+	+	+	+	+
Skret-Magierlo, J.E. 2014 [41]	18% (9/50)	/	NS	+	NS	+	+	NS	NS	NS	/	/
Wei, Y.S. 2016 [40]	16% (33/206)	/	/	+	+	+	+	/	+	+	/	+
Zhu, Y. 2018 [37]	8.57% (18/210)	OS ↓	DFS ↓	/	/	+	+	+	+	+	/	+
Tang, M. 2019 [35]	10.59% (43/406)	OS ↓	DFS ↓	NS	/	NS	+	/	+	+	+	/
Wan, T. 2021 [36]	8.8% (162/1836)	OS ↓	PFS ↓	/	/	/	+	+	/	+	+	+
Wei, W.W. 2022 [38]	12.1% (21/174)	OS ↓	DFS ↓	/	/	/	/	/	/	/	/	/
Chen, X.L. 2024 [39]	22.6% (273/1208)	OS ↓	DFS ↓	NS	/	NS	+	+	+	+	NS	+

OS, overall survival; DFS, disease-free survival; PFS, progression-free survival; NS, no significance; “+” represents a statistically significant correlation; “/” represents not mentioned in the study.

## Supplementary Materials

The following supporting information can be downloaded at: https://www.mdpi.com/article/10.3390/diagnostics14141517/s1, Figure S1: Volcano plot of differentially expressed genes associated with the PNI in cervical cancer patients in the TCGA cohort. MUC2 was strongly upregulated in the PNI (+) group (log2 fold-change = 3.15, *p* < 0.001).

## Abbreviations

TME	tumor microenvironment
PNI	perineural invasion
NSRH	nerve-sparing radical hysterectomy
RH	radical hysterectomy
SHP	superior hypogastric plexus
IHP	inferior hypogastric plexus
TH	tyrosine hydroxylase
NPY	neuropeptide Y
VIP	vasointestinal peptide
OS	overall survival
DFS	disease-free survival
PFS	progression-free survival
H&E	hematoxylin and eosin
IHC	immunohistochemistry
SCs	Schwann cells
AUC	area under the receiver operating characteristic curve
NTs	neurotrophins
CNTFs	ciliary neurotrophic factors
GDNFs	glial cell-derived neurotrophic factors
NGF	nerve growth factor
BDNF	brain-derived neurotrophic factor
NT-3	neurotrophin 3
NT-4/5	neurotrophin 4/5
NT-6	neurotrophin 6
EMT	Epithelial–mesenchymal transition
MMPs	matrix metalloproteinases
SP	substance P
CGRP	calcitonin gene-related peptide
GFAP	glial fibrillary acidic protein
PACAP	pituitary adenylate cyclase-activating polypeptide
NMB	neuromedin B
MUC1	membrane-bound mucin 1
MAG	myelin-associated glycoprotein
