# Supplementary material for: Perineural Invasion in Cervical Cancer: A Hidden Trail for Metastasis
Source: Diagnostics (Basel). 2024 Jul 14;14(14):1517. doi: 10.3390/diagnostics14141517 (PMC11275432; doi:10.3390/diagnostics14141517)
Supplement: Supplementary file 1 [file diagnostics-14-01517-s001.zip › diagnostics-3021950-supplementary.pdf]

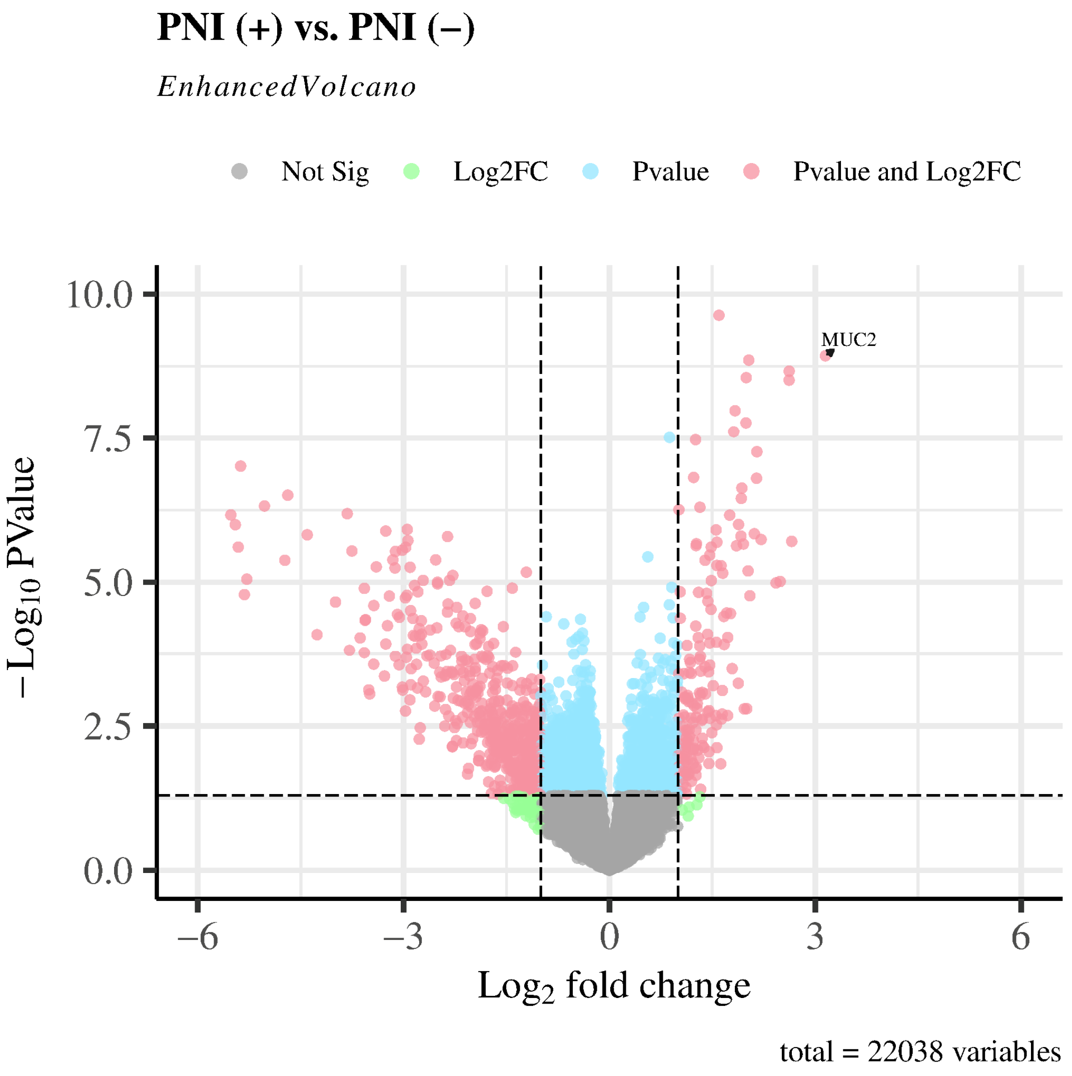

Supplementary Figure S1. Volcano plot of differentially expressed genes associated with the PNI in cervical cancer patients in the TCGA cohort. MUC2 was strongly upregulated in the PNI (+) group (log2 fold-change = 3.15, $P < 0.001$).
